# Erythropoietin inhibits chemotherapy-induced cell death and promotes a senescence-like state in leukemia cells

**DOI:** 10.1038/s41419-018-1274-6

**Published:** 2019-01-08

**Authors:** Thuc-Nghi Duc Pham, Weili Ma, David Miller, Lidia Kazakova, Samuel Benchimol

**Affiliations:** Department of Biology, York University, Toronto, ON M3J 1P3 Canada

## Abstract

There are conflicting reports on the adverse effects of erythropoietin (EPO) for the management of cancer-associated anemia. The recognition that erythropoietin receptors (EPORs) are expressed outside the erythroid lineage and concerns that erythropoiesis-stimulating agents (ESAs) may cause tumors to grow and increase the risk of venous thromboembolism have resulted in substantially fewer cancer patients receiving ESA therapy to manage myelosuppressive chemotherapy. In this study, we found that EPO suppresses p53-dependent apoptosis induced by genotoxic (daunorubicin, doxorubicin, and γ-radiation) and non-genotoxic (nutlin-3a) agents and induces a senescence-like state in myeloid leukemia cells. EPO interferes with stress-dependent Mdm2 downregulation and leads to the destabilization of p53 protein. EPO selectively modulates the expression of p53 target genes in response to DNA damage preventing the induction of a number of noncoding RNAs (ncRNAs) previously associated with p53-dependent apoptosis. EPO also enhances the expression of the cyclin-dependent kinase inhibitor p21^WAF1^ and promotes recruitment of p53 to the p21 promoter. In addition, EPO antagonizes Mcl-1 protein degradation in daunorubicin-treated cells. Hence, EPO signaling targets Mcl-1 expression and the p53-Mdm2 network to promote tumor cell survival.

## Introduction

The p53 tumor suppressor protein coordinates the cellular response to stress in mammalian cells. Basal levels of p53 are low primarily due to interaction with the Mdm2 E3 ubiquitin ligase that mediates degradation of p53. In response to diverse stress signals, including DNA damage, telomere shortening, and oncogene-induced replicative stress, p53 protein undergoes extensive posttranslational modification resulting in increased stability and activity^1^. Once activated, p53 protein functions primarily as a sequence-specific DNA binding transcription factor to regulate the expression of genes and noncoding RNAs (ncRNAs) that collectively contribute to p53-dependent cellular responses including apoptosis, cell cycle arrest, senescence, and DNA repair. The divergent biological outcomes mediated by p53 are thought to be due to differential transcription of p53 target genes^2,3^. The targeting of p53 to different promoters is influenced by many factors, including p53 protein levels, posttranslational modifications of p53 that regulate its interaction with various transcriptional coactivators, the specific p53 response element sequence, and the intrinsic properties of diverse p53 core promoters that affect binding affinity and p53 recruitment^1–5^.

Erythropoietin (EPO), a glycoprotein produced in the kidney under hypoxic conditions, functions as the principal regulator of red blood cell production by controlling the proliferation, survival, and differentiation of immature erythroid progenitors into mature red cells. Upon binding EPO, the EPO receptor (EPOR) undergoes dimerization that in turn activates the receptor-associated tyrosine kinase, Janus Kinase 2 (JAK2). Activated JAK2 phosphorylates tyrosine residues found on the cytosolic domain of the EPOR leading to the recruitment of downstream effectors, including PI3K, GRB2, and the STAT family members^6–9^.

Previously, we reported that EPO protects DP16.1/p53ts cells from p53-dependent apoptosis^10^. DP16.1/p53ts cells were derived by stable expression of a temperature-sensitive (ts) p53 allele (A135V) in the p53-null, spleen focus-forming virus-transformed, mouse erythroleukemia cell line DP16.1. DP16.1/p53ts cells grow well at 37 °C and undergo p53-dependent apoptosis when p53 is activated at 32 °C. At 32 °C, in the presence of EPO, DP16.1/p53ts cells remain viable and arrest in the G1 phase of the cell cycle^10^. Numerous extracellular cytokines, including EPO, IL3, IL6, macrophage migration inhibitory factor (MIF) and stem cell factor (SCF), have been shown to prevent p53-dependent apoptosis^11–18^. The common ability of survival-promoting cytokines to suppress p53-induced apoptosis may reflect a physiological mechanism through which p53-positive tumors gain resistance to apoptosis-inducing anticancer agents^19^.

Erythropoiesis-stimulating agents (ESAs), including EPO, were used routinely to treat anemia in cancer patients receiving myelosuppressive chemotherapy. ESAs increase red blood cell production in bone marrow by activating the EPOR on erythroid progenitor cells resulting in a decreased need for red blood cell transfusion. EPO and its receptor, however, are expressed in various tissues outside the hematopoietic system with tissue protective effects of EPO demonstrated initially in the brain, heart and kidney^20,21^. In 2003, two studies found that patients with metastatic breast cancer and patients with head and neck cancer who received recombinant human EPO (rHuEPO) in combination with chemotherapy or radiation therapy to manage cancer-associated anemia exhibited higher mortality compared with patient groups who received a placebo^22,23^. Subsequent clinical studies reported that the use of ESAs to treat cancer patients reduced overall survival possibly related to an increased risk of thromboembolism and increased tumor progression^24–30^. The ongoing concern that ESAs may be linked to increased mortality risks has resulted in substantially fewer cancer patients receiving ESA therapy to manage myelosuppressive chemotherapy^31^ and remains highly controversial^32–34^.

Here we examine the ability of EPO to protect DA3/EPOR murine leukemia cells from stress-induced apoptosis. These EPOR-expressing cells express wild-type p53 and undergo apoptosis in response to genotoxic stress. They provide an experimental model to investigate the effect of EPO on cancer cells exposed to chemotherapy. We demonstrate that EPO destabilizes p53 protein, selectively modulates p53-target gene expression, increases Mcl-1 protein expression and promotes a senescence-like state that protects DA3/EPOR cells from genotoxic and non-genotoxic stress.

## Materials and methods

### Cell culture

DA3/EPOR murine leukemic cells^35^ were maintained at 37 °C with 5% CO_2_ in RPMI 1640 media supplemented with 10% fetal bovine serum (FBS) and 1 U/ml recombinant human erythropoietin (EPO). OCI-M1 human acute myeloid leukemic cells^36^ were maintained at 37 °C with 5% CO_2_ in α-MEM with 10% FBS.

### Drug treatment

DA3/EPOR cells were washed with RPMI 1640 containing 10% FBS and incubated at 37 °C for 1 h without EPO. Cells are then treated with daunorubicin (DNR) (0.25 µM) or doxorubicin (Dox) (200 ng/ml), in the absence or presence of rHuEPO (1 U/ml). Cell viability was assessed by staining with trypan blue. MG-132 was purchased from Sigma-Aldrich and added directly to cells in culture media at a final concentration of 5 μM. Nutlin-3a was purchased from Santa Cruz (sc-45061).

### Cell cycle analysis

Cells were pelleted and washed with phosphate buffered saline (PBS) and fixed in cold 75% ethanol before freezing at −20 °C until use. Fixed cells were washed with cold PBS at 4 °C and suspended in Staining Buffer (0.2% Triton X-100, 1 mM ethylenediaminetetraacetic acid [EDTA], in PBS) followed by incubation with RNaseA (100 µg/ml, ThermoScientific) at 37 °C for 30 min. Cells were stained with 50 µg/ml propidium iodide for 1 h. Propidium iodide fluorescence was measured using a BD FACSCalibur flow cytometer (Becton Dickinson) and data analysis was performed using the BD CellQuest Pro software.

### Nucleofection

Nucleofection was performed according to the manufacturer’s instructions (Lonza). DA3/EPOR cells (2×10^6^) were suspended in Cell Line Nucleofector Solution V and combined with 2 µg of plasmid vector comprised of a 9:1 ratio of pCMV/p53DD plasmid^37^ to pSUPER-puromycin or PCDNA3 (Invitrogen Life Technology). Cells were pulsed with the Nucleofector X-001 program using a Nucleofector 2b device. Pulsed cells were transferred to prewarmed media containing 10% FBS and EPO (1 U/ml) and allowed to recover for 48 h. Clones were selected in puromycin (1 µg/ml, Sigma-Aldrich Canada Ltd.) over 4 weeks. p53DD expression was confirmed by immunoblotting.

### Apoptosis assays

Caspase-3 activity was measured cells using the NucView 488 Caspase-3 Assay Kit according to the manufacturer’s instructions (Biotium, Inc.). TUNEL assay was performed as described^15^.

### Northern blotting

Total RNA was isolated and RNA samples were run on a denaturing agarose gel for coding RNAs or acrylamide gel for ncRNAs. Hybridization of radioactive probes was performed using standard conditions.

### Western blotting

Cells were directly lysed in 1× SDS (sodium dodecyl sulfate) lysis buffer (10% SDS, 10% glycerol, 88 mM Tris-HCl pH 6.8, in water) and boiled at 97 °C for 10 min followed by addition of 0.1% bromophenol blue and 0.1 M dithiothreitol (DTT). Total protein was separated by SDS-polyacrylamide gel electrophoresis and transferred onto nitrocellulose membranes followed by incubation with primary antibodies. The antibodies to p53 included: PAb421^38^, FL393 (sc-6243, Santa Cruz Biotechnology), DO-1 (sc-126, Santa Cruz Biotechnology). Antibodies to Mdm2 included: SMP14 (sc-965; Santa Cruz Biotechnology), p-MDM2 (Ser166) (#3521; Cell Signaling Technology), and HDM2-323 (sc-56154; Santa Cruz Biotechnology). Antibodies to p21 (sc-397), JAK2 (sc-278), pJAK2 (sc-21870), Akt-1 (sc-5298), p-Akt1 (sc-293125), and β-actin were from Santa Cruz Biotechnology. Anti-MCL1 antibody [Y37] (ab32087) was from Epitomics/Abcam. Protein levels were measured using a Typhoon Trio Plus Imager (GE Healthcare) with Cy3 and Cy5-conjugated secondary antibodies or visualized using HRP-conjugated secondary antibodies with enhanced chemiluminescence detected on film.

### Cycloheximide treatment

After treatment of cells with DNR and/or EPO, 40 µg/ml cycloheximide (Sigma-Aldrich) was added directly to culture media to stop new protein synthesis. Cells were harvested at different times after cycloheximide addition. p53 and β-actin protein levels were determined by quantitative immunoblotting with detection on a Typhoon Trio Plus Imager (GE Healthcare) and ImageJ software. Relative p53 expression was normalized to β-actin levels and plotted relative to initial p53 levels in the absence of cycloheximide addition.

### Quantitative real-time PCR

Total RNA was extracted from cells using TRIzol (Life Technologies) according to the manufacturer’s instructions. cDNA synthesis was performed with 2 μg of total RNA using the High Capacity RNA-to-cDNA Kit (ThermoFisher Scientific) and subjected to PCR using the SsoFaster EvaGreen Supermix Kit and an Applied Biosystems 7500 Real-Time PCR System (ThermoFisher Scientific) according to the manufacturer’s instructions. Relative expression was calculated using the 2^−∆∆Ct^ method; Ct values were normalized to 18S RNA using the Pfaffl method^39^. Primer efficiencies were evaluated and calculated prior to use in qRT-PCR. The following oligonucleotides were used as qRT-PCR primers:

*Pri-miR-34a*^40^, F 5′ CTG TGC CCT CTT GCA AAA GG 3′, R 5′ GGA CAT TCA GGT GAG GGT CTT G 3′;

*Pri-miR-34b/c*, F 5′ CTC GGT TTG TAG GCA GTG TA 3′, R 5′ TTG ATG GCA GTG GAG TTA GTG 3′;

*lincRNA-p21*, F 5′ CAT TCC GTC TCC AGT TCC TAA C 3′, R 5′ CGA AGA GAC AAC GGC ACA CTT 3′;

*PIDD*, F 5′ TCC AGC AAG ATG TGA GCT TAT G 3′, R 5′ GGT CAT TCC AGG TGT TAC T 3′;

*PUMA*, F 5′ CGG AGA CAA GAA CC AGC AAG ATG TGA GCT TAT G 3′, R 5′ GGT CAT TCC AGG TGT TAC T 3;

*NOXA*, F 5′ TCG CAA AAG AGC AGG ATG AG 3′, R 5′ CAC TTT GTC TCC AAT CCT CCG 3′;

*p21*, F 5′ CCA GAC ATT CAG AGC CAC AGG 3′, R CGA AGA GAC AAC GGC ACA CTT 3′;

*p53*, F 5′ TAG GTA GCG ACT ACA GTT AGG G 3′, R 5′ CAT GGC AGT CAT CCA GTC TT 3′;

*Mcl-1*, F 5′ CAA AGA GGC TGG GAT GGG TTT-3′, R 5′ CCC TAT TGC ACT CAC AAG GC 3′;

*18S*, F 5′ GTG TTG AGG AAA GCA GAC AT 3′, R 5′ CAG TCT GGG ATC TTG TAC TG 3′.

### ChIP-qPCR

ChIP (chromatin immunoprecipitation) assays were performed as described^41,42^ with the following modifications. After formaldehyde fixation, cells were pelleted at 1200 RPM at 4 °C for 8 min and washed consecutively with: (i) ice-cold PBS (Corning), (ii) 0.25% Triton X-100, 10 mM EDTA (pH 8.0), 0.5 mM ethylene glycol tetraacetic acid (EGTA) (pH 7.5), 10 mM Hepes (pH 7.5) containing protease inhibitor cocktail tablet (Roche) and (iii) 12.5 mM NaCl, 1 mM EDTA (pH 8.0), 0.5 mM EGTA (pH 7.5), 10 mM Hepes containing protease inhibitor cocktail tablet. Cells were resuspended in lysis buffer (0.15 M NaCl, 25 mM Tris pH 7.5, 5 mM EDTA pH 8.0, 1% Triton X-100, 0.1% SDS, 0.5% Sodium deoxycholate, protease inhibitor cocktail tablet) and the DNA was sonicated to 100–400 bp using a Sonic Dismembrator Model 500 (ThermoFisher Scientific). Sonicated cell lysates were centrifuged at 14,000 RPM at 4 °C to pellet cell debris. Cell extracts were precleared with prewashed Protein-A agarose beads (Bioshop Canada Inc.) for 1 h at 4 °C.

Cell extracts containing 1 mg of protein were incubated with 2 µg of antibody (FL393 or control IgG) at 4 °C overnight. Forty micrograms of protein from precleared cell lysates were used as input. Cell extracts were incubated with 40 µl of prewashed Protein-A agarose beads for 1 h at 4 °C and pelleted at 4000 RPM for 5 min at 4 °C. Protein-Agarose bead complexes were washed consecutively with: (i) RIPA buffer (150 mM NaCl, 50 mM Tris pH 8.0, 0.1% SDS, 0.5% Sodium deoxycholate, 1% NP-40, protease inhibitor cocktail), (ii) high salt solution (0.5 M NaCl, 50 mM Tris pH 8.0, 0.1% SDS, 1% NP-40, protease inhibitor cocktail), (iii) LiCl solution (0.25 M LiCl, 50 mM Tris pH 8.0, 0.5% Sodium deoxycholate, 1% NP-40, protease inhibitor cocktail), and (iv) TE buffer (10 mM Tris pH 8.0, 1 mM EDTA pH 8.0, protease inhibitor cocktail). Washed beads were then incubated with IP elution buffer (2% SDS, 10 mM DTT, 0.1 M NaHCO_3_) for 30 min at room temperature. Eluted protein complexes were collected and boiled at 65 °C overnight after addition of 20 µl of 4 M NaCl to reverse crosslinks.

One microliter of RNaseA (10 mg/ml) was added to each sample and incubated at 37 °C for 30 min. Twenty microliters of 10× proteinase K buffer (0.1 M Tris-HCl pH 8.0, 0.05 M EDTA pH 8.0) and 20 µl of proteinase K (20 mg/ml) was added to each reaction and incubated at 42 °C for 1 h. DNA was then separated from each reaction via phenol-chloroform extraction and recovered by ethanol precipitation. Samples were pelleted at 14,000 RPM for 15 min at 4 °C and DNA pellets were washed with 80% ethanol and stored in RNase/DNase free water at −80 °C until use.

ChIP DNA samples were subjected to quantitative real-time PCR using SsoFaster EvaGreen Supermix Kits and an Applied Biosystems 7500 Real-Time PCR System (ThermoFisher Scientific) according to the manufacturer’s instructions. Ct values were normalized to non-IP input samples for each treatment and expressed as enrichment compared with input. The following oligonucleotides were used as ChIP-qPCR primers:

*p21*, F 5′ ACCAGCAGCAAAATCGGAGC 3′, R 5′ CCCACAGCTGGTAGTTGGGTATC 3′.

### Statistical tests

Data are presented as mean values ± standard error of the mean. *P* values were determined by the two-tailed Student’s *t* test (to compare the means of two different samples) or by a one-way ANOVA (to compare more than two groups), followed by a Tukey’s multiple comparison test using Microsoft Excel and SPSS software.

## Results

### EPO suppresses DNA damage-induced apoptosis in DA3/EPOR cells

**Fig. 1:**
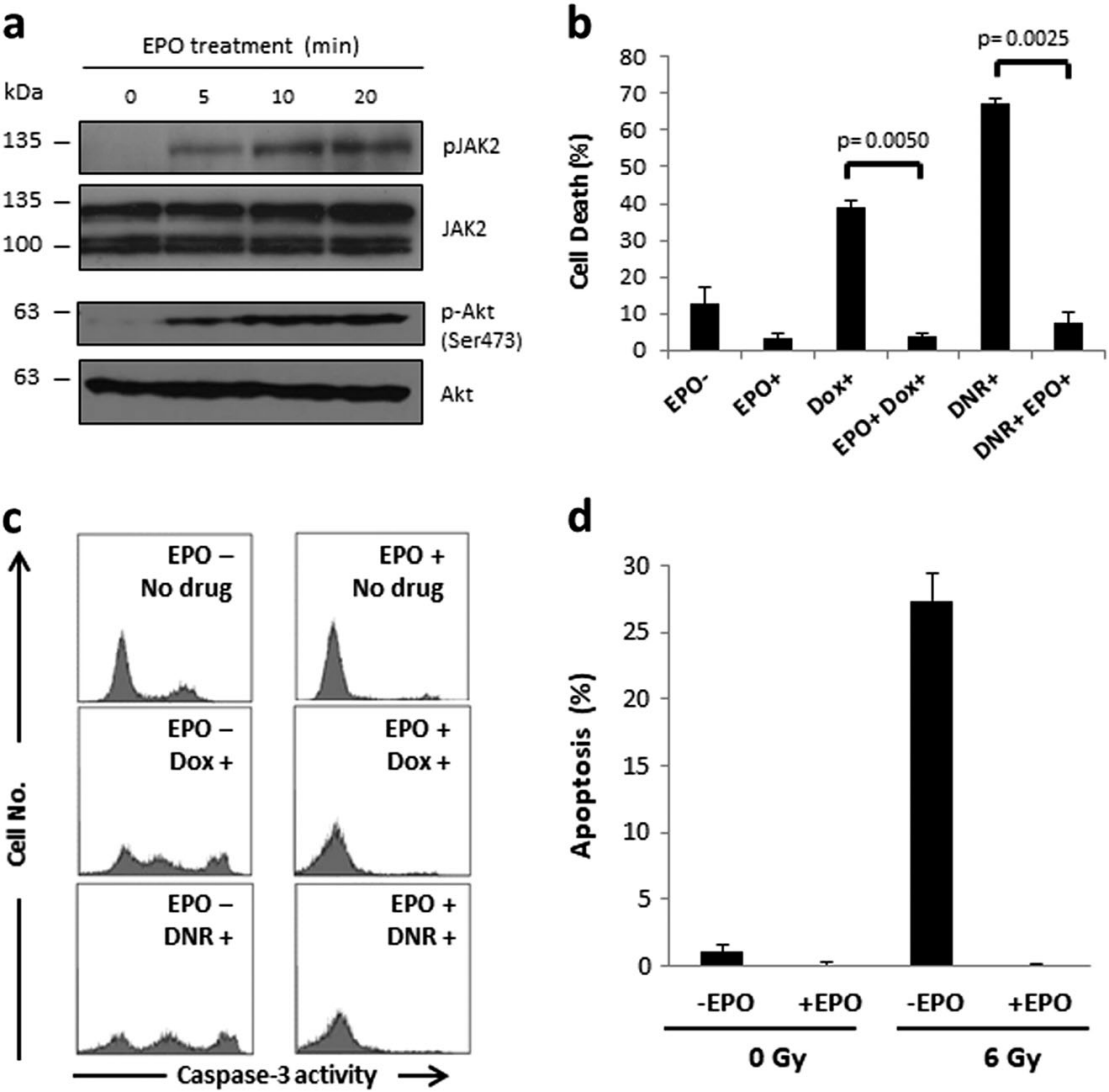
EPO suppresses DNA damage-induced apoptosis in DA3/EPOR cells. **a** DA3/EPOR cells were cultured in the absence of EPO for 1.5 h and then stimulated with EPO (1 U/ml) for the times indicated. JAK2 and AKT kinase activation was determined by western blot analysis using a phospho-specific Tyr 1007/Tyr 1008 antibody (pJAK2) and phospho-specific Ser 473 antibody (pAKT) respectively. **b** Cells were cultured in the absence of EPO for 1 h followed by 6 h of treatment with Dox (200 ng/ml) or DNR (0.25 µM), with or without EPO (1 U/ml). The proportion of apoptotic cells (cells with <2 N DNA content) was determined by flow cytometry after propidium iodide staining. Error bars represent SEM (*N* = 3 biological replicates) and *p* values were obtained via Student’s two-tailed *t* test. **c** Cells were cultured in the absence of EPO for 1.5 h, followed by 20 h treatment with Dox (200 ng/ml) or DNR (0.25 µM) in the presence or absence of EPO (1 U/ml). Caspase-3 activity was measured by flow cytometry. **d** Cells were cultured in the absence of EPO for 1.5 h, exposed to 6 Gy γ-radiation and cultured for an additional 6 h in the presence or absence of EPO (1 U/ml) before assessment of apoptosis by TUNEL assay. Error bars represent SEM (*N* = 3 biological replicates)

DA3/EPOR cells were derived from the IL-3-dependent DA3 murine leukemic cell line after transfection with wild-type EPOR cDNA^35^. We have determined that these cells express wild-type p53 based on cDNA sequence analysis. To confirm that these cells are responsive to EPO, we measured the phosphorylation of two downstream effectors in the EPO signaling pathway, JAK2 and Akt. EPO-induced phosphorylation of JAK2 (Tyr1007/Tyr1008) and Akt (Ser473) was detected within 5 min of treatment and increased with time (Fig. 1a). These data indicate that DA3/EPOR cells are responsive to EPO and may be used as an experimental model to assess how EPO influences the response of leukemia cells to DNA-damaging agents. DA3/EPOR cells undergo apoptosis in response to doxorubicin (Dox), daunorubicin (DNR) and γ-irradiation. Treatment of these cells with EPO effectively prevents drug- and radiation-induced cell death (Fig. 1b–d).

### EPO promotes cell viability but not cell proliferation

**Fig. 2:**
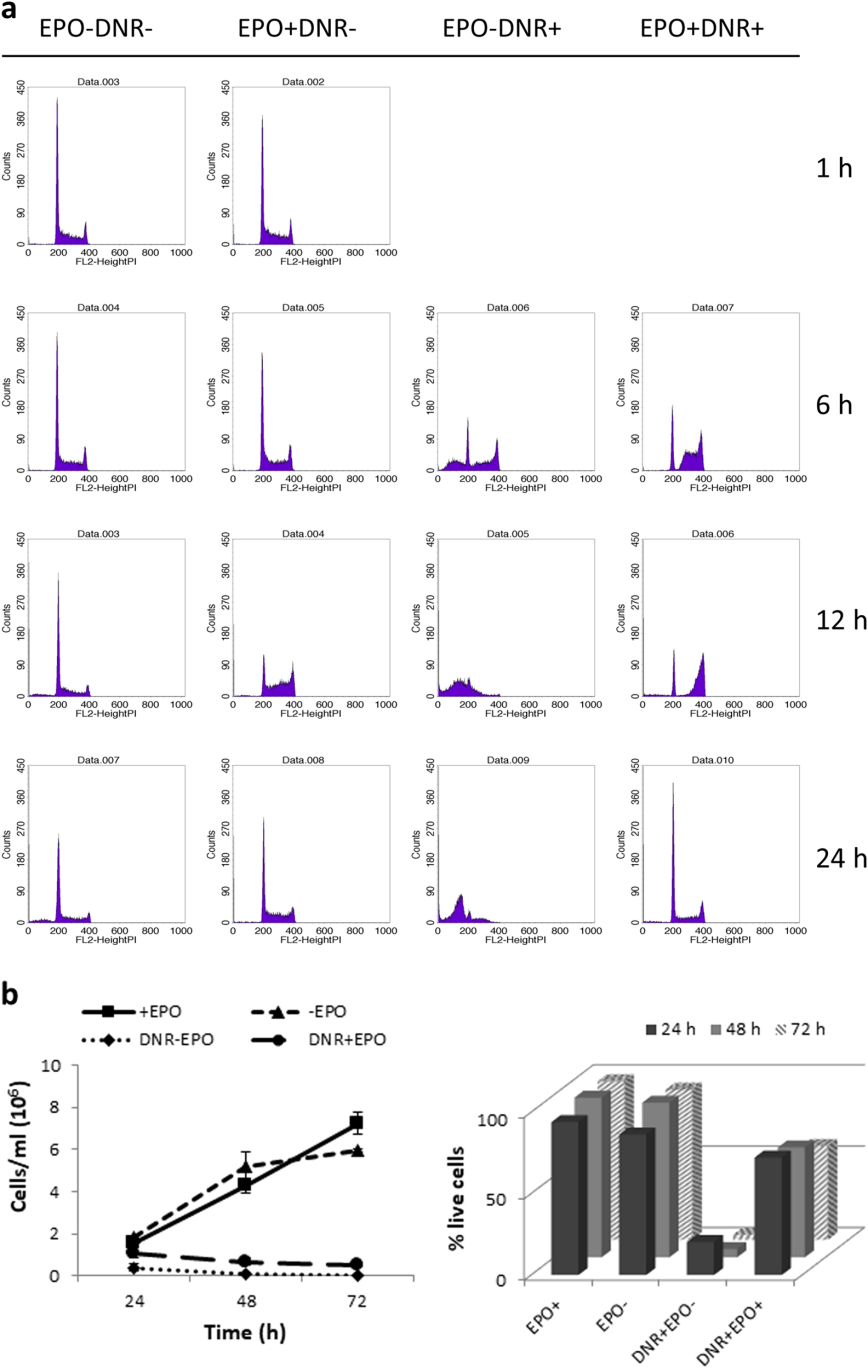
EPO promotes sustained arrest in G1. **a** Cells were cultured in the absence of EPO for 1 h followed by treatment with DNR (0.25 µM) in the presence or absence of EPO (1 U/ml) for the times indicated. Treated cells were fixed with 75% ethanol and then stained with propidium iodide prior to analysis by flow cytometry. Apoptotic populations of cells have <2 N DNA content. Representative cell cycle profiles are shown. **b** DA3/EPOR cells were left untreated or treated with DNR (0.25 µM) in the presence or absence of EPO (1 U/ml) for 24 h. DNR was withdrawn and the cells were placed in medium supplemented with 10% FBS and EPO (1 U/ml). At the times indicated, viable cell numbers were determined by Trypan blue staining. The right panel shows the proportion of live cells in each of the four samples at the times indicated

DA3/EPOR cells treated with DNR and EPO for 24 h show an accumulation of nonapoptotic cells in the G1 phase of the cell cycle (Fig. 2a). To determine if these cells retain proliferative capacity, DA3/EPOR cells were treated with DNR in the presence or absence of EPO for 24 h. The cells were then washed to remove DNR and placed in drug-free medium containing 10% FBS and EPO for 24, 48, and 72 h prior to cell counting (Fig. 2b). Similar to cells treated with DNR in the absence of EPO for 24 h, cells treated with DNR and EPO for 24 h did not proliferate when placed in normal medium despite a large proportion of cells remaining viable (Fig. 2b, right panel). These findings indicate that EPO promotes survival of DA3/EPOR cells that have undergone genotoxic stress and that these cells are incapable of undergoing further cell division.

### DA3/EPOR cells undergo p53-dependent apoptosis in response to DNR

To determine if DNR-induced cell death is dependent on p53 in DA3/EPOR cells, we generated three subclones expressing the C-terminal fragment of p53 (p53DD). This fragment, consisting of amino acids 302–390 of p53, lacks the DNA binding and transactivation domains of p53 and retains the oligomerization domain. p53DD acts as a potent trans-dominant repressor of wild-type p53 through its ability to prevent the formation of functional p53 tetramers^37^. Endogenous p53 protein levels are high in DA3/EPOR cells expressing p53DD, likely resulting from the formation of stable heterologous oligomers (Fig. S1). DA3/EPOR-DD clones exhibit reduced apoptosis in response to DNR compared with parental cells (Fig. S1). EPO retained the ability to reduce apoptosis in the DNR-treated DA3/EPOR-DD clones (Fig. S1). These data indicate that DNR induces p53-dependent apoptosis in DA3/EPOR cells that can be rescued by EPO.

### EPO modulates the level of Mcl-1 protein in DNR-treated cells

**Fig. 3:**
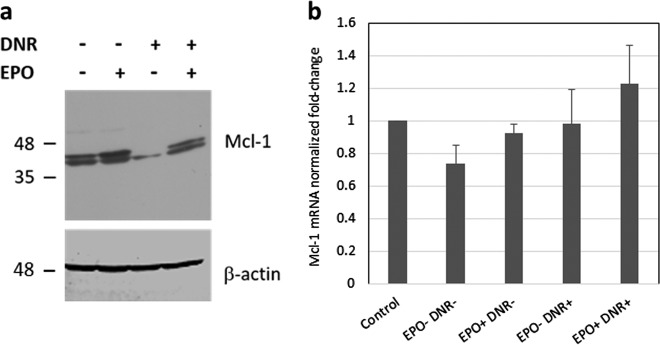
EPO promotes Mcl-1 protein expression in DNR-treated cells. **a** DA3/EPOR cells were cultured in the absence of EPO for 6 h followed by 6 h of treatment with DNR (0.25 µM) in the presence or absence of EPO (1 U/ml). Mcl-1 protein expression was determined by western blot analysis. β-actin levels serve as a loading control. The blot shown is representative of two independent experiments. **b** RT-qPCR analysis of Mcl-1 RNA. RNA was prepared from cells treated as in (**a**). Ct values were normalized to 18S rRNA. Error bars represent SEM (*N* = 3 biological replicates). One-way ANOVA did not return a significant f-statistic. Control cells were cultured in complete medium and were not treated with DNR

The prosurvival protein, Mcl-1, is associated with resistance to anticancer therapies and its expression is usually maintained by cytokines in hematopoietic cells. In addition, a number of DNA-damaging agents have been reported to reduce Mcl-1 protein levels by promoting its degradation^43^. Hence, we investigated the expression of Mcl-1 in DA3/EPOR cells treated with DNR in the presence or absence of EPO. In untreated cells, Mcl-1 appears as a doublet by immunoblot analysis likely resulting from proteolytic processing^44^. DNR treatment reduced Mcl-1 levels and EPO restored Mcl-1 expression (Fig. 3a). These changes in Mcl-1 protein expression were not reflected by Mcl-1 mRNA levels (Fig. 3b) indicating that DNR likely triggers Mcl-1 protein degradation and that this process is antagonized by EPO post-transcriptionally. The degradation of Mcl-1 is considered an important early event in apoptosis and may be required to initiate the apoptotic response^43,45^. The ability of EPO to modulate Mcl-1 protein expression likely contributes to cell survival.

### EPO promotes p53 destabilization in DNR-treated cells

**Fig. 4:**
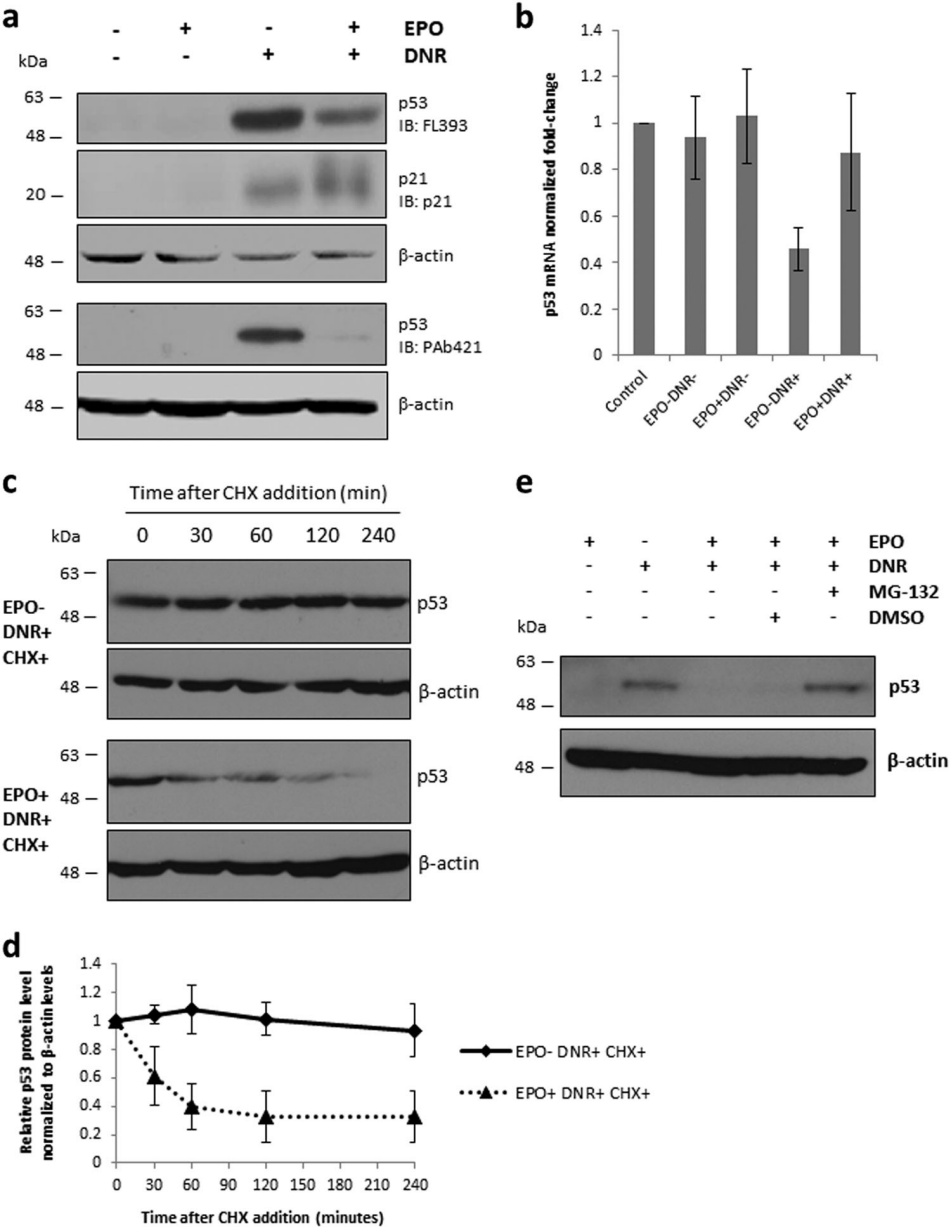
EPO promotes p53 destabilization through a posttranslational mechanism. **a** Cells were cultured in the absence of EPO for 1 h followed by 6 h of treatment with DNR (0.25 µM) in the presence or absence of EPO (1 U/ml). Protein expression levels were determined by western blot analysis using the indicated antibodies. The FL393 blot was stripped and re-probed with a p21 antibody. β-actin protein levels were used as a loading control for the FL393/p21 blot and for a separate PAb421 blot. **b** RT-qPCR analysis of p53 RNA reveals that p53 mRNA remained unaltered after EPO treatment. RNA was prepared from cells treated as in (**a**). Ct values were normalized to 18S rRNA using the Pfaffl method. Error bars represent SEM (*N* = 3 biological replicates). **c** Cells were cultured in the absence of EPO for 1 h followed by 10 h of treatment with DNR (0.25 µM) in the presence or absence of EPO (1 U/ml). Cycloheximide (40 µg/ml) was added at the 6 h time point of treatment for a total of 4 h. Cells were harvested at the times indicated and p53 expression was measured by western blot analysis. β-actin protein levels were used as a loading control. Protein bands were visualized using fluorochrome-conjugated secondary antibodies and detected using a Typhoon variable mode imager. The data shown are representative of three independent experiments. **d** Densitometric analyses of relative p53 proteins levels were performed using the ImageJ program and normalized to β-actin levels for each time point. Normalized p53 protein levels were then plotted as a relative value compared with normalized p53 protein levels at 0 min after cycloheximide addition (*N* = 3 biological replicates). **e** Cells were cultured in the absence of EPO for 1 h followed by 10 h of treatment with DNR (0.25 µM) with or without EPO (1 U/ml). MG-132 (5 µM) was added at the 6 h time point for a total of 4 h. p53 protein levels were measured by western blot analysis. A representative western blot is shown

Next, we measured the levels of p53 protein and mRNA in DA3/EPOR cells treated with DNR in the presence or absence of EPO. As expected, p53 protein levels increased in DNR-treated cells, likely resulting from posttranslational stabilization of p53 protein (Fig. 4a). Western immunoblotting revealed a decrease in p53 protein expression in cells treated with DNR and EPO compared with DNR alone. The decrease was detected with a polyclonal antibody (FL393) and with a monoclonal antibody (PAb421) directed to p53 (Fig. 4a). The decrease in p53 protein expression was not accompanied by any decrease in p53 mRNA, consistent with a post-transcriptional mechanism (Fig. 4b). Expression of the cell cycle arrest protein p21, an important transcriptional target of p53^46^, increased in response to DNR treatment and remained elevated in cells treated with DNR and EPO (Fig. 4a).

To determine whether EPO destabilizes p53 protein, DA3/EPOR cells were treated with cycloheximide to inhibit protein synthesis and the amount of p53 protein in cell extracts was measured by western blotting. Cells were treated with DNR for 10 h in the presence or absence of EPO. Cycloheximide was added during the last 4 h of treatment. In the absence of EPO, p53 protein levels remained stable in DNR-treated cells (Fig. 4c, d). In the presence of EPO, there was a significant destabilization of p53 protein in DNR-treated cells. Consistent with our finding that EPO destabilizes p53 in DNR-treated cells, the addition of the proteasome inhibitor MG-132 completely prevented the decrease in p53 levels by EPO (Fig. 4e). Taken together, these data indicate that EPO destabilizes p53 protein in DNR-treated cells through the proteasomal degradation pathway.

### EPO regulates the Mdm2−p53 pathway

**Fig. 5:**
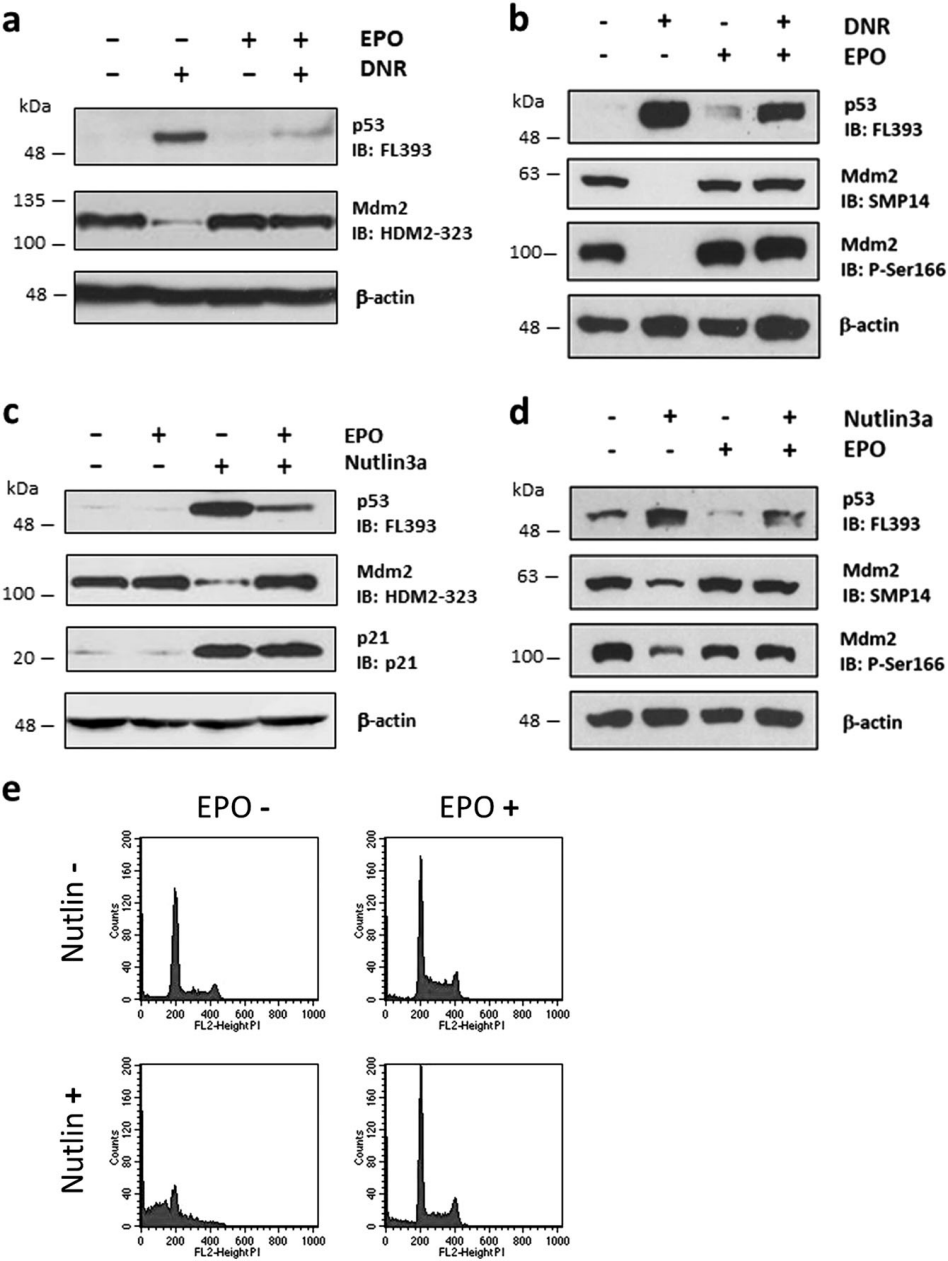
EPO increases Mdm2 expression after DNR or nutlin-3a treatment and suppresses nutlin-3a-induced apoptosis. Western blot showing p53 and Mdm2 protein levels in cells treated with DNR (**a**, **b**) or with nutlin-3a (**c**, **d**) in the presence or absence of EPO. In (**a**) and (**b**), DA3/EPOR cells were cultured in the absence of EPO for 1 h followed by 10 h of treatment with DNR (0.25 µM) with or without EPO (1 U/ml). In (**c**) and (**d**), cells were cultured in the absence of EPO for 0.5 h followed by 24 h of treatment with nutlin-3a (10 µM) with or without EPO (1 U/ml). Protein expression levels were determined by western blot analysis using the indicated antibodies. The HDM2-323 antibody recognizes full-length MDM2 and the SMP14 antibody recognizes the MDM2 p60 cleavage product. (**e**) Cells were treated as in (**c**, **d**) and the proportion of cells undergoing apoptosis (cells having <2 N DNA content) was measured by flow cytometry after staining with propidium iodide

An important regulator of p53 protein stability is the Mdm2 E3 ubiquitin ligase^1^. The stabilization of p53 protein following DNA damage is believed to result from stress-induced modifications in p53 and Mdm2 that disrupt p53-Mdm2 binding. To determine if EPO modulates Mdm2 expression, we measured the levels of p53 and Mdm2 protein in DA3/EPOR cells treated with DNR in the presence or absence of EPO. There was a decrease in Mdm2 protein levels following DNA damage and this reduction was prevented by EPO (Fig. 5a). The reduction of Mdm2 expression following DNA damage and the ability of EPO to counteract this effect were confirmed with two additional antibodies directed to Mdm2 (Fig. 5b). The downregulation of Mdm2 in response to DNA damage was reported previously and has been attributed to enhanced Mdm2 degradation due to phosphorylation^47,48^ or epitope masking due to phosphorylation^49^. Uniform results with three antibodies to Mdm2 renders the second possibility less likely. The ability of EPO to interfere with DNR-dependent Mdm2 downregulation and restore Mdm2 expression in DNR-treated cells is likely to contribute to p53 protein destabilization.

In the next series of experiments, we used nutlin-3a, a small molecule antagonist of Mdm2 that stabilizes and activates p53 through a non-genotoxic process^50^. Treatment of DA3/EPOR cells with nutlin-3a for 24 h elevated p53 protein levels and decreased Mdm2 protein levels (Fig. 5c) as was seen previously following DNR treatment. The reduction of Mdm2 expression following nutlin-3a treatment was confirmed with two additional Mdm2 antibodies (Fig. 5d). Nutlin-3a induced apoptosis in a large fraction of the cells (Fig. 5e). EPO attenuated all these effects by nutlin-3a: Mdm2 levels were restored to normal, p53 expression was suppressed and apoptosis was largely prevented (Fig. 5c–e). We found that Mdm2 is constitutively phosphorylated on Ser166 in DA3/EPOR cells, and that EPO restored Mdm2 expression and phosphorylation (Ser166) in both DNR-treated and nutlin-3a-treated cells (Fig. 5a–d). Phosphorylation of Mdm2 at Ser166 is associated with Mdm2 protein stabilization in unstressed cells^51^. p21 protein levels increased in response to nutlin-3a treatment and remained elevated in cells treated with nutlin-3a and EPO (Fig. 5c) as seen previously with DNR in Fig. 4a. The ability of EPO to suppress nutlin-3a-mediated apoptosis and to interfere with the nutlin-3a-mediated changes in p53 and Mdm2 levels suggests that EPO signaling regulates the Mdm2−p53 interaction.

### EPO does not protect mutant p53-expressing OCI-M1 cells from DNR-induced apoptosis

Since EPO protects against DNR-induced apoptosis in p53 wild-type cells through a process that involves p53 destabilization, we reasoned that EPO should have no effect on mutant p53-expressing cells. We selected the OCI-M1 human erythroleukemia cell line to test this idea because these cells express stable mutant p53 protein (L145R) and EPORs^36,52^. We confirmed that OCI-M1 cells are responsive to EPO stimulation by measuring the phosphorylation of JAK2, the receptor-proximal effector of EPO signaling (Fig. S2a). We then treated OCI-M1 cells with DNR in the absence or presence of EPO and measured p53 protein levels and apoptosis. OCI-M1 cells express high levels of mutant p53 protein that were unaffected by DNR or EPO (Fig. S2b). Moreover, DNR-mediated apoptosis in OCI-M1 cells was unaffected by EPO (Fig. S2c). These results indicate that EPO does not enhance survival of EPO-responsive, mutant p53-expressing OCI-M1 cells.

### EPO selectively modulates the expression of p53 target genes

After p53 activation, numerous p53 target genes are upregulated to coordinate the cellular response to stress. The ability of EPO to reduce p53 protein levels and to protect cells from DNR-, Dox-, γ-radiation- and nutlin-3a-induced cell killing raises the possibility that EPO might affect the p53 transcriptional program. For example, low-affinity consensus binding sites on p53 target genes might not bind to p53 when p53 levels are low^53^. Moreover, Mdm2, in addition to regulating p53 degradation, binds to the N-terminal transcriptional activation domain of p53 to inhibit its transcriptional activity. Mdm2 colocalizes with p53 protein on the promoters of certain p53 responsive genes^54^ and hence, Mdm2 may exert a selective influence on p53 target gene expression through its interaction with p53 protein bound to chromatin^3,55^. To investigate this possibility, we examined the expression of three proapoptotic genes, *Puma, Pidd*, and *Noxa*, and several noncoding RNAs (miR-34a, miR-34b/c, and lincRNA-p21) that are transcriptional targets of p53. The miR-34 family represses genes that are required for proliferation and cell survival and ectopic expression of miR-34 leads to growth inhibition or apoptosis^56,57^. lincRNA-p21 has been shown to play a role in p53-dependent apoptosis after DNA damage^58^.

**Fig. 6:**
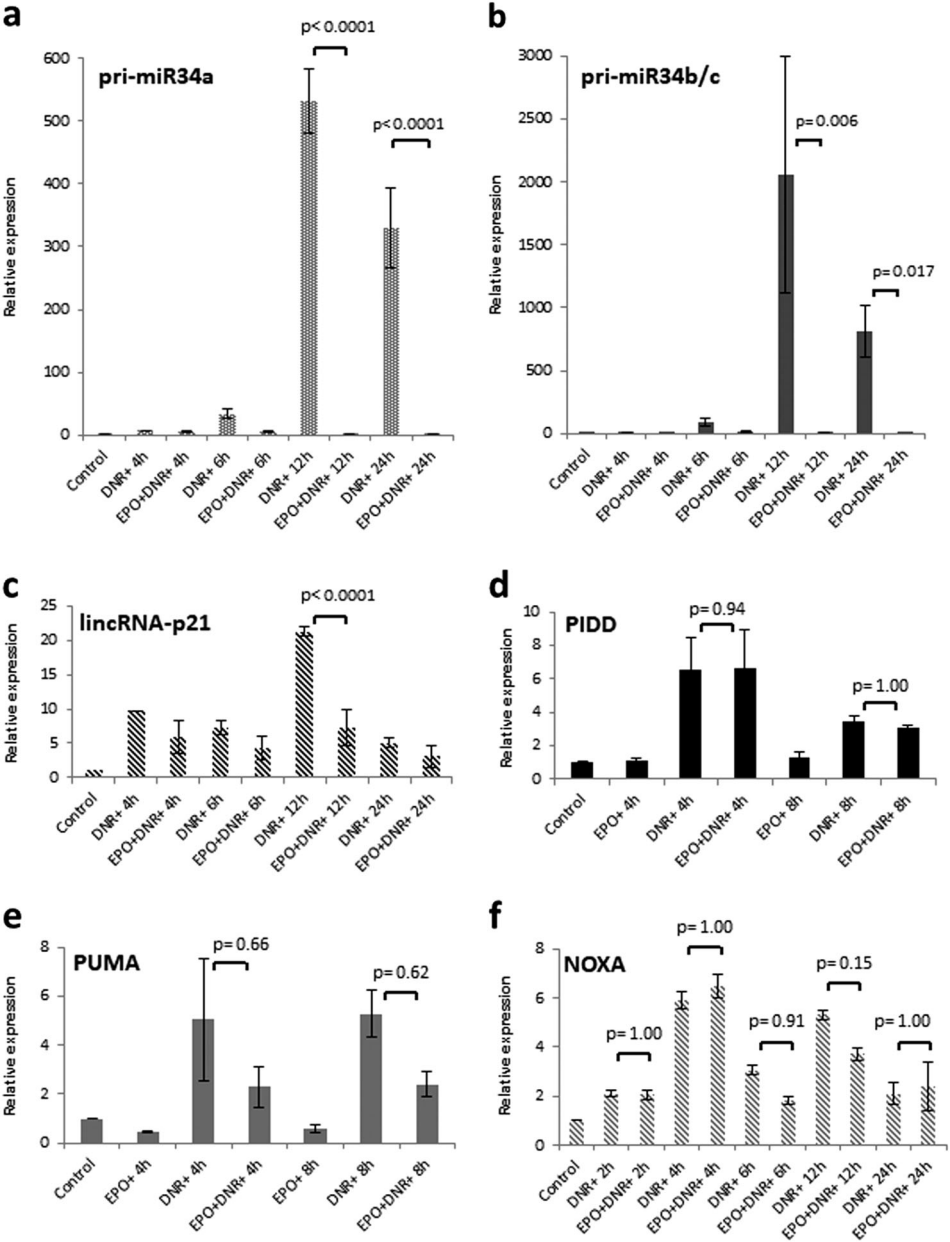
EPO selectively modulates the expression of DNR-induced p53 target genes. Real-time PCR (qRT-PCR) analysis of p53 target gene expression in DA3/EPOR cells treated with DNR (0.25 μM) in the presence or absence of EPO (1 U/ml) for different periods. Cells were cultured in the absence of EPO for 1 h followed by treatment with DNR with or without EPO. Ct values were normalized to control 18S rRNA using the Pfaffl method. Error bars represent SEM (*N* = 3 biological replicates). Statistical analysis of the results was evaluated by one-way ANOVA followed by Tukey’s HSD post hoc test

**Fig. 7:**
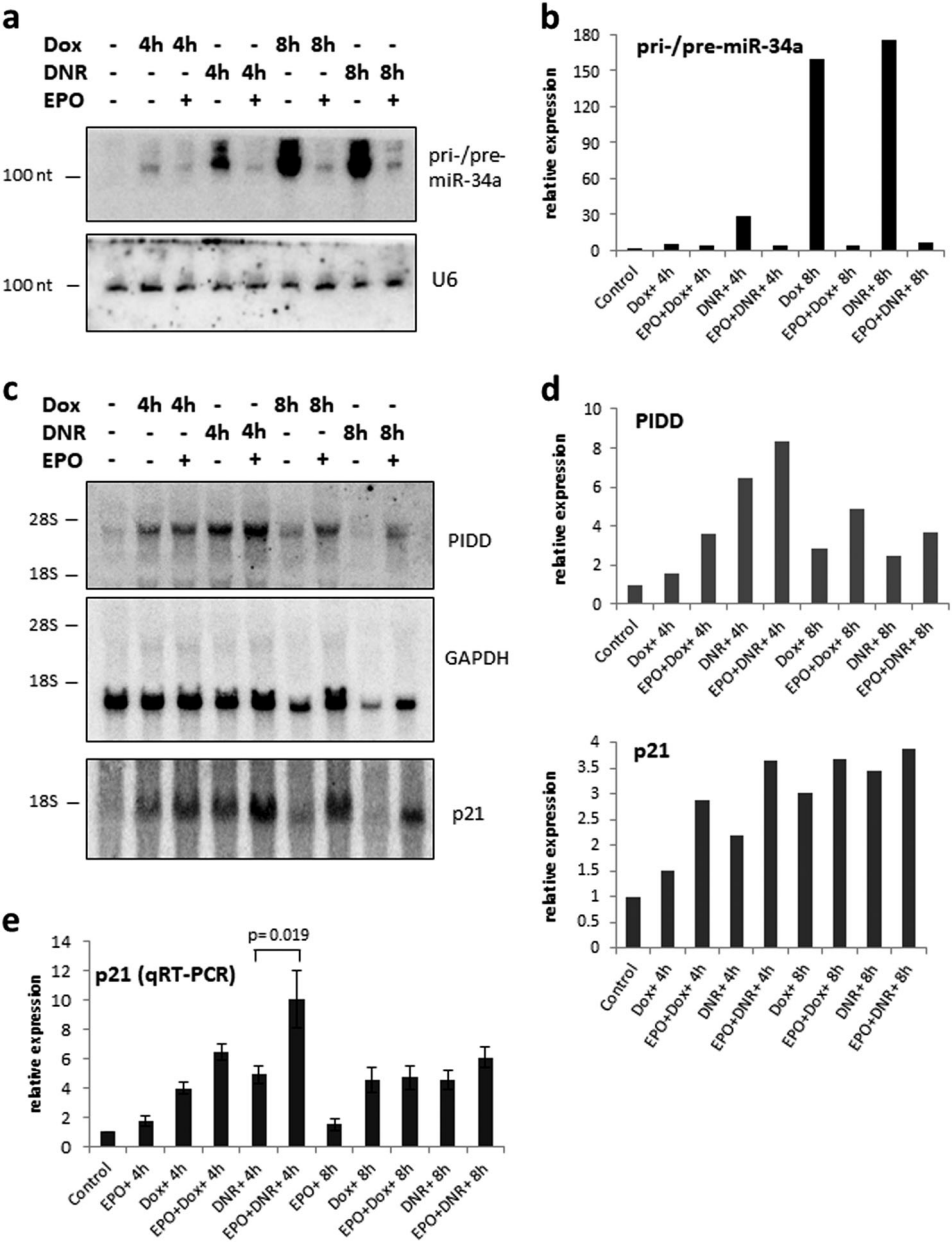
EPO selectively modulates the expression of drug-induced p53 target genes. **a** Northern blot analysis showing pri-/pre-miR-34a expression in DA3/EPOR cells treated with Dox (200 ng/ml) or DNR (0.25 µM) in the presence or absence of EPO. U6 served as the loading control for miR-34a. **b** Relative abundance of pri-/pre-miR-34a was determined by phosphor-image analysis after normalizing to the level of U6 mRNA in each sample. **c** Northern blot analysis showing Pidd and p21 mRNA expression in DA3/EPOR cells treated with Dox (200 ng/ml) or DNR (0.25 µM) in the presence or absence of EPO (1 U/ml). The same filter was hybridized sequentially and GAPDH served as the loading control. **d** Relative abundance of Pidd and p21 expression was determined by phosphor-image analysis after normalizing to the level of GAPDH mRNA in each sample. **e** qRT-PCR analysis of p21 mRNA expression in DA3/EPOR cells. Cells were cultured in the absence of EPO for 1 h followed by treatment with Dox (200 ng/ml) or DNR (0.25 µM) with or without EPO (1 U/ml) for 4 and 8 h. Ct values were normalized to 18S rRNA using the Pfaffl method. Error bars represent SEM (*N* = 3 biological replicates) and *p* values were obtained via one-way ANOVA followed by Tukey’s HSD post hoc test

Pri-miR-34a, pri-miR-34b/c, and lincRNA-p21 were strongly induced at late times (12 h) after drug treatment and their induction was significantly suppressed by EPO (Fig. 6a–c). In contrast, drug-induced expression of *Puma, Noxa*, and *Pidd* was not significantly affected by EPO (Fig. 6d–f). The EPO-sensitivity of pri-miR-34a expression and EPO-insensitivity of *Pidd* expression after drug treatment was confirmed by northern blot analysis (Fig. 7a–d).

### EPO increases p21 expression and p53 recruitment to the p21 promoter despite decreased p53 protein levels

Next, we examined drug-induced p21^WAF1^ RNA expression. EPO influences the cellular response to p53 activation by promoting cell survival and the accumulation of cells in G1; both of these processes are regulated by p21. We had not seen an effect of EPO on p21 protein expression after DNR treatment (Fig. 4a) or nutlin-3a treatment (Fig. 5c). p21 mRNA was measured by northern blotting (Fig. 7c, d) and qRT-PCR (Fig. 7e). As expected, p21 RNA expression increased within 4 h of drug (DNR, Dox) treatment. We observed a further increase in p21 expression when DA3/EPOR cells were treated with drug in the presence of EPO (Fig. 7c, d). The qRT-PCR data confirmed that cells treated with DNR and EPO expressed more p21 RNA than cells treated with DNR alone at the 4-h time point but not at the 8-h time point (Fig. 7e). Taken together, these data suggest that EPO enhances drug-induced p21 mRNA expression in DA3/EPOR cells.

**Fig. 8:**
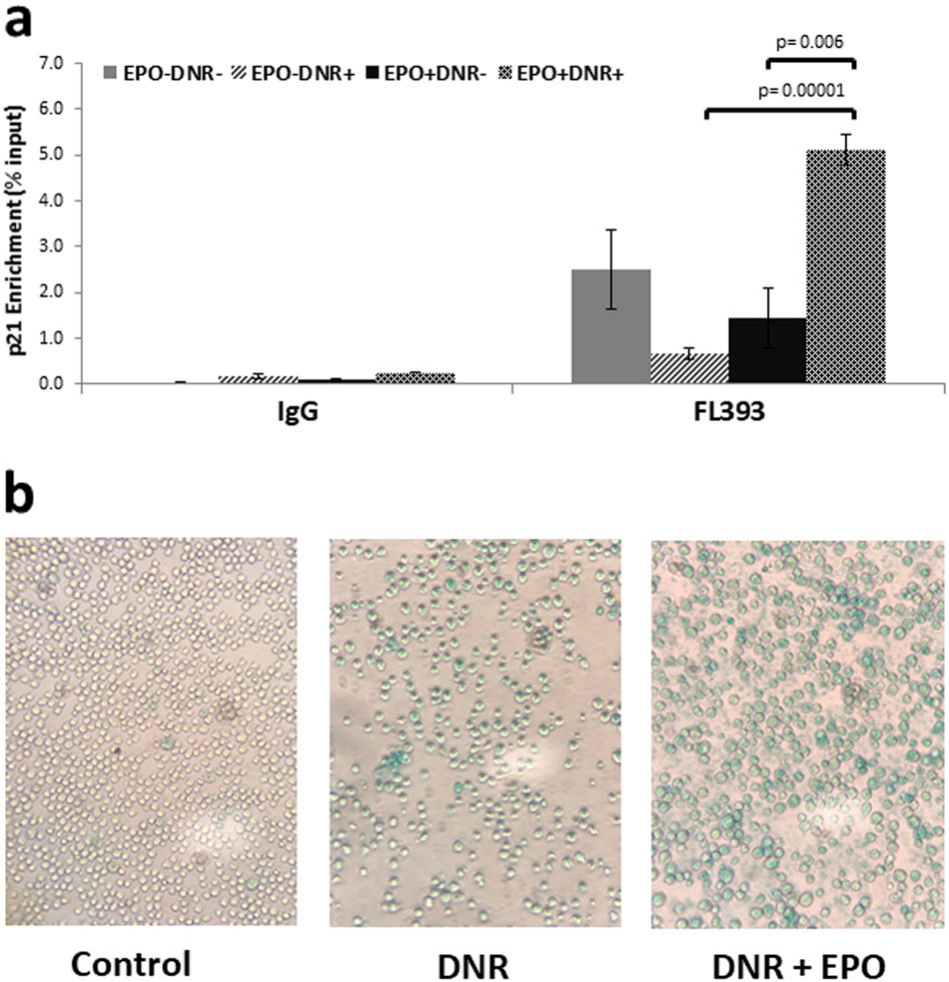
EPO enhances recruitment of p53 to the p21 promoter and sustains a senescence-like state. **a** Chromatin immunoprecipitation coupled with quantitative PCR (ChIP-qPCR) was used to evaluate p53 binding to the p21 promoter. Cells were cultured in the absence of EPO for 1 h followed by 16 h of treatment with DNR (0.25 μM) in the presence or absence of EPO (1 U/ml). Ct values were normalized to non-IP input and p53 levels for each sample, and are expressed as Enrichment (% input). Error bars represent SEM (*N* = 3 biological replicates) and *p* values were obtained from one-way ANOVA followed by Tukey’s HSD post hoc test. **b** DA3/EPOR cells were treated with DNR in the presence or absence of EPO for 24 h. The cells were then washed to remove DNR and placed in drug-free medium containing 10% FBS and EPO for an additional 24 h prior to staining for SAβgal as described^60^. Control cells were cultured in complete medium and were not treated with DNR. The images were taken at ×200 magnification

We used ChIP-qPCR to determine if EPO influenced the recruitment of p53 to the p21 promoter in DNR-treated DA3/EPOR cells. DA3/EPOR cells were treated with DNR in the absence or presence of EPO for 16 h, and then harvested for ChIP-qPCR (Fig. 8a). p53 enrichment at the p21 promoter was normalized to total p53 protein levels in each sample. p53 protein was present at the p21 promoter under nonstress conditions and this basal level of p53 at the promoter did not change in cells treated with EPO alone or with DNR alone. This is consistent with previous reports showing that p53 is present on target promoters in unstressed cells and becomes transcriptionally active only after stress stimulation^59^. We found a significant increase in p53 recruitment to the p21 promoter in cells treated with DNR and EPO compared with cells treated with EPO alone or DNR alone.

### EPO promotes a senescence-like state

We have shown that cells treated with EPO and DNR are viable, do not proliferate, express sustained elevated levels of p21^WAF1^, and accumulate in the G1 phase of the cell cycle. This is reminiscent of cellular senescence, a process associated with aging in which cells enter a state of permanent cell cycle arrest. To investigate the possibility that cells treated with DNR and EPO enter a senescence-like state, we examined senescence-associated β-galactosidase (SAβgal) activity, a widely used senescence biomarker^60^. As expected, we noticed fewer surviving cells remaining after DNR treatment compared with the untreated control cells. We found positive SAβgal staining in the remaining DNR-treated cells (Fig. 8b) consistent with previous studies showing that cancer cells can undergo senescence in response to treatment with selected anticancer compounds or radiation in a process known as therapy-induced senescence^61^. SAβgal staining was detected in nearly all cells treated with DNR and EPO but not in the untreated control cells (Fig. 8b).

## Discussion

In this study, we show that EPO promotes the survival of DA3/EPOR myeloid leukemia cells treated with genotoxic and nongenotoxic agents. The ability of EPO to promote survival has been attributed to various processes including EPOR upregulation^62^, JAK2 kinase activation^13,15,29^, PI3K/Akt pathway activation^62,63^, Erk phosphorylation^30,64,65^, Bim phosphorylation and degradation^66^, GSK-3β downregulation^62^ and expression of antiapoptotic proteins^67–69^. Many of these EPO-dependent changes are cell type-specific. The ability of EPO to activate several intracellular signaling pathways downstream of EPOR ligation that individually promote survival may explain the widespread protective effects of EPO on various tissues and cells.

We find that EPO destabilizes p53 protein after DNR and nutlin-3a treatment. Moreover, we show a correlation between p53 and Mdm2 expression. DNR and nutlin-3a enhance p53 protein expression and decrease Mdm2 expression. EPO counteracts these effects of DNR and nutlin-3a resulting in decreased p53 protein levels and increased Mdm2 protein levels. Nutlin-3a treatment was initially shown to increase the level of Mdm2 protein expression^50^ in contrast to what we see in DA3/EPOR cells. We confirmed the downregulation of Mdm2 expression following nutlin-3a or DNR treatment using three different antibodies directed to Mdm2. It is possible that Mdm2-nutlin-3a complexes exhibit different stability in different cell types. Our findings suggest a model in which EPO promotes survival of DA3/EPOR cells after treatment with genotoxic and nongenotoxic agents in part through increased Mdm2-dependent degradation of wild-type p53 protein.

In addition, we find that DNR reduces the level of the antiapoptotic Mcl-1 protein and that EPO restores Mcl-1 protein expression in cells treated with DNR. Mcl-1 is an unstable protein with a short half-life and its expression is regulated by various growth factors. The degradation of Mcl-1 in response to various DNA-damaging agents is considered to be an important early event in apoptosis^43,45^. We suggest that both the upregulation of Mcl-1 expression and the downregulation of p53 expression may contribute to the prosurvival function of EPO in drug-treated cells.

We find that EPO selectively influences p53 target gene expression. EPO enhances p21 expression, represses the expression of pri-miR34a, pri-miR34b/c and lincRNA-p21, and has no effect on drug-induced *Pidd, Puma* and *Noxa* expression. The repression of pri-miR-34a and pri-miR-34b/c cannot be attributed to enhanced degradation of these ncRNAs since both were found to be stable in DNR-treated DA3/EPOR cells and EPO had no effect on pri-miRNA half-life when the DNR-treated cells were maintained in medium containing actinomycin D for 8 h to inhibit transcription (data not shown).

We do not know if the changes in p53 target gene expression resulting from EPO treatment contribute to EPO-mediated cell survival or are a consequence of EPO treatment. For example, we cannot exclude the possibility that the suppression of pri-miR-34a, pri-miR34b/c, and lincRNA-p21 in response to EPO treatment reflects the accumulation of cells in the G1 phase of the cell cycle. In one recent study, various human cells were sorted by fluorescence-activated cell sorting and miRNA expression was found to be stable throughout the cell cycle^70^. No common pattern of ncRNA expression during the cell cycle has emerged^71,72^.

We have shown that cells treated with EPO and DNR enter a senescence-like state. Recent studies show that senescent cells acquire a senescent-associated secretory phenotype (SASP), which involves the secretion of proinflammatory cytokines that promote cancer progression^73^. In a mouse breast cancer model, Jackson et al. reported that p53 wild-type tumors, unlike mutant p53 tumors, undergo senescence in response to chemotherapy, avoiding abnormal mitoses and cell death^74^. The senescent tumor cells acquired an SASP that could induce proliferation in neighboring, nonsenescent cells. The tumor cells of cancer patients receiving chemotherapy or radiation treatment will undergo excessive stress leading to cell death or senescence. The administration of ESAs to control chemotherapy-induced anemia could redirect the cellular stress response towards senescence and tumor cell survival, especially in p53 wild-type tumors. Senescent cells could exert deleterious effects on the tissue microenvironment through the secretion of proinflammatory cytokines and could contribute to tumor progression and clinical relapse. Our findings speak to the safety concerns related to the use of ESAs to treat cancer-associated anemia.

## Supplementary information

Supplementary Figures

Supplementary figure legends
