# Supplementary material for: Erythropoietin inhibits chemotherapy-induced cell death and promotes a senescence-like state in leukemia cells
Source: Cell Death Dis. 2019 Jan 8;10(1):22. doi: 10.1038/s41419-018-1274-6 (PMC6325163; doi:10.1038/s41419-018-1274-6)
Supplement: Supplementary file 1 — Supplementary Figures [file 41419_2018_1274_MOESM1_ESM.pptx]

## Slide 1
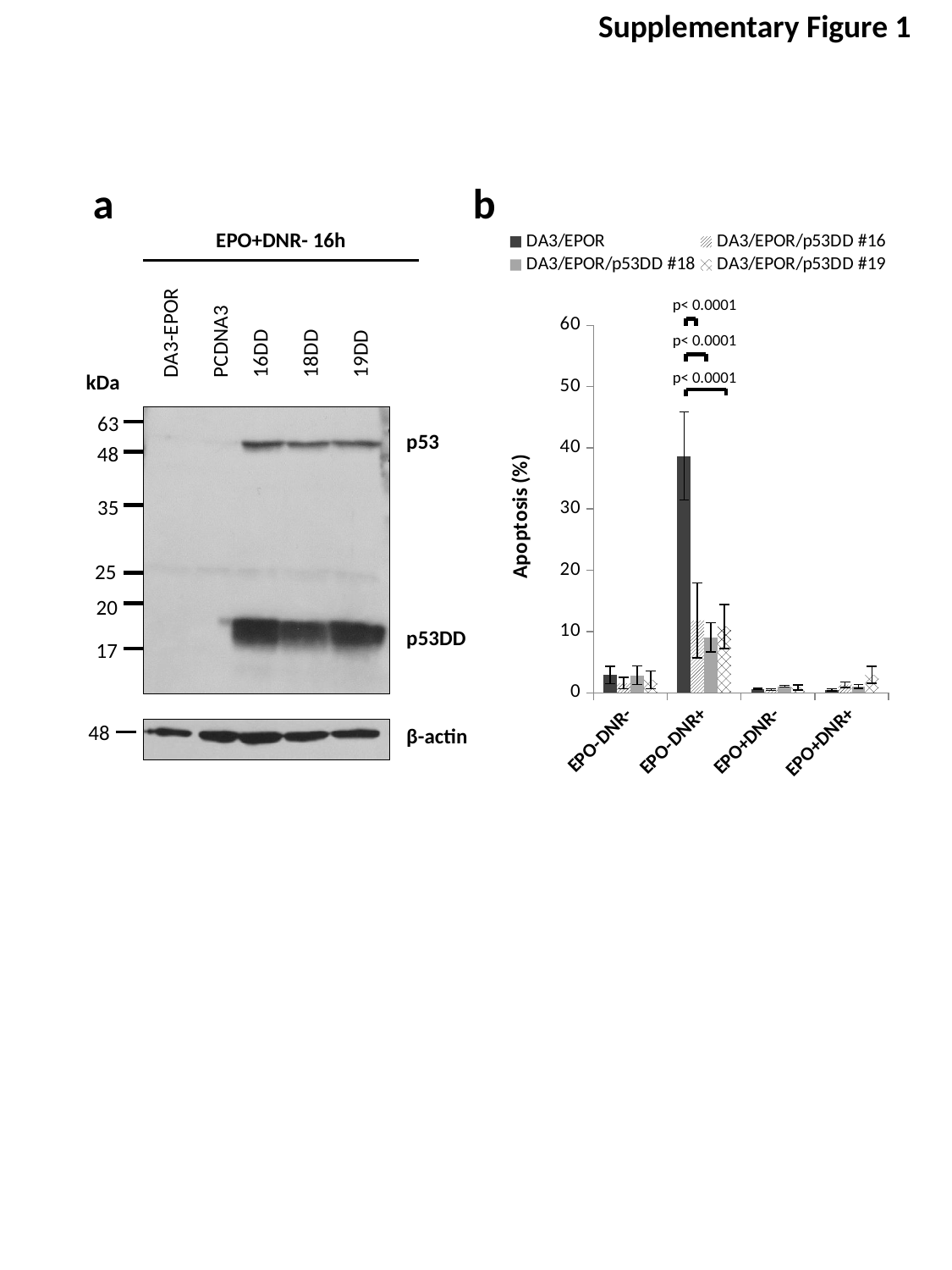

Supplementary Figure 1
### Chart
| Category | DA3/EPOR | DA3/EPOR/p53DD #16 | DA3/EPOR/p53DD #18 | DA3/EPOR/p53DD #19 |
|---|---|---|---|---|
| EPO-DNR- | 2.893333333333333 | 1.5733333333333333 | 2.856666666666667 | 2.123333333333333 |
| EPO-DNR+ | 38.666666666666664 | 11.819999999999999 | 9.066666666666668 | 10.806666666666667 |
| EPO+DNR- | 0.6433333333333333 | 0.52 | 1.0633333333333335 | 0.8666666666666667 |
| EPO+DNR+ | 0.5 | 1.3233333333333335 | 1.0333333333333332 | 2.91 |p< 0.0001
p< 0.0001
p< 0.0001
a
b
EPO+DNR- 16h
DA3-EPOR
PCDNA3
19DD
16DD
18DD
kDa
63
p53
48
35
25
20
p53DD
17
β-actin
48

## Slide 2
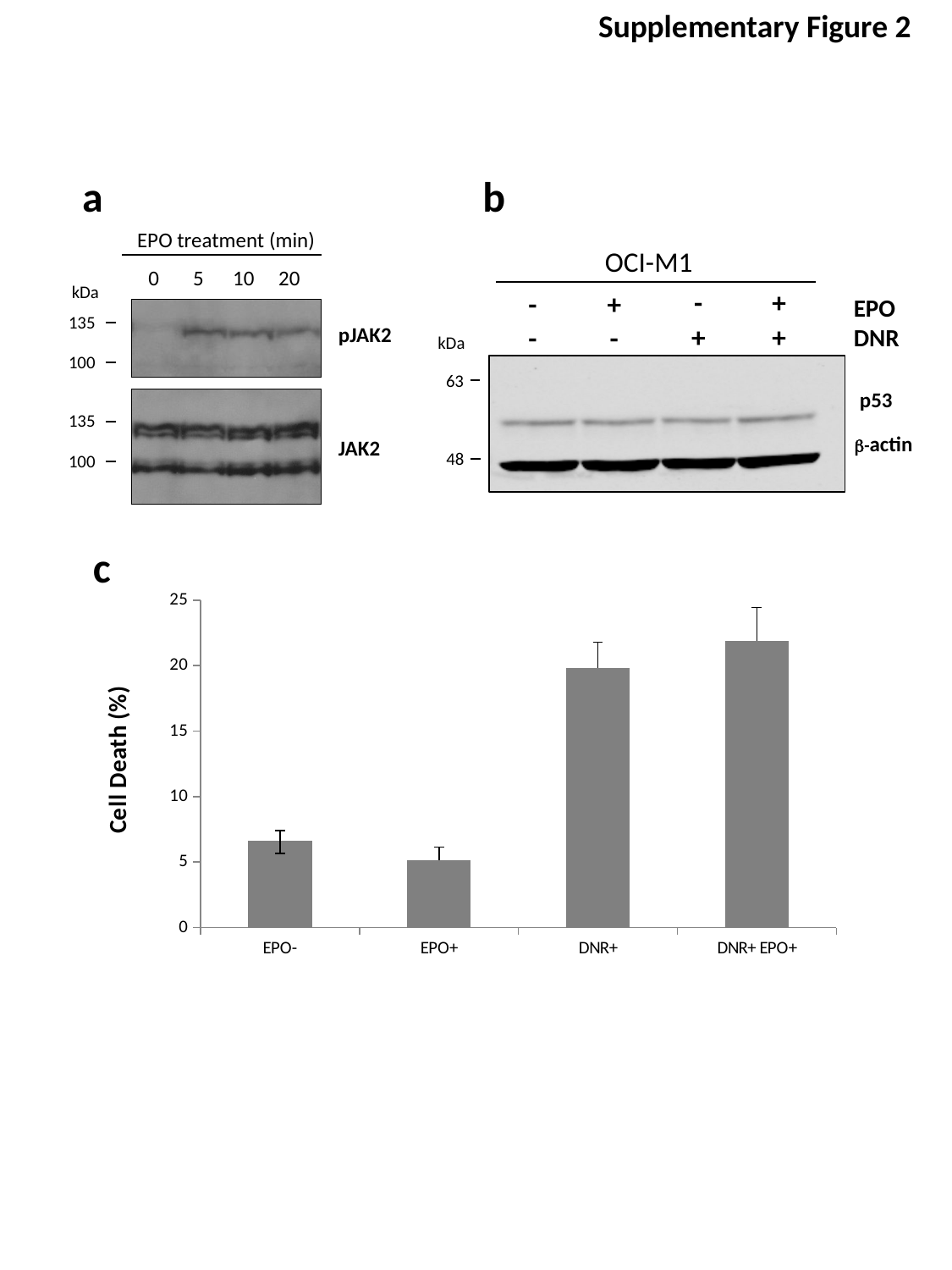

Supplementary Figure 2
a
b
OCI-M1
-
+
+
+
-
-
+
-
EPO
DNR
kDa
 63
p53
β-actin
 48
EPO treatment (min)
 0 5 10 20
kDa
135
pJAK2
100
135
100
JAK2
c
### Chart
| Category | |
|---|---|
| EPO- | 6.646666666666666 |
| EPO+ | 5.17 |
| DNR+ | 19.856666666666666 |
| DNR+ EPO+ | 21.89 |
