# Supplementary material for: Erythropoietin inhibits chemotherapy-induced cell death and promotes a senescence-like state in leukemia cells
Source: Cell Death Dis. 2019 Jan 8;10(1):22. doi: 10.1038/s41419-018-1274-6 (PMC6325163; doi:10.1038/s41419-018-1274-6)
Supplement: Supplementary file 2 — Supplementary figure legends [file 41419_2018_1274_MOESM2_ESM.docx]

**Supplementary Figure Legends**

**Supplementary Fig. 1. DA3/EPOR cells undergo p53-dependent apoptosis after treatment with DNR. (a)** DA3/EPOR cells were co-nucleofected with pCMV/p53DD and pSUPER-puromycin vectors or a PCDNA3 control. 19 clones were selected after puromycin selection over four weeks. Clones 16, 18, and 19 displayed the strongest expression of p53DD as determined by Western blot analysis using PAb421 monoclonal antibody that recognizes a C-terminal domain on p53. β-actin protein levels were used as a loading control. **(b)** DA3/EPOR/p53DD clones and DA3/EPOR parental cells were cultured in the absence of EPO for 1 h followed by 16 h of treatment with DNR (0.25 µM) in the presence or absence EPO (1 U/ml). Treated cells were fixed with 75% ethanol and then stained with propidium iodide. The proportion of apoptotic cells (with < 2 N DNA content) was determined by flow cytometry after propidium staining. Error bars represent SEM; p-values were determined by one-way ANOVA followed by Tukey’s HSD post hoc test (N=3 biological replicates).

**Supplementary Fig. 2. EPO does not suppress DNR-induced apoptosis in mutant p53-expressing OCI-M1 cells despite EPOR activation. (a)** Western blot showing JAK2 activation in OCI-M1 cells following EPO treatment. OCI-M1 cells were cultured in the absence of EPO and then stimulated with EPO (1 U/ml) for the times indicated. JAK2 activation was measured using a phospho-specific Tyr 1007/Tyr 1008 antibody (pJAK2). **(b)** Western blot showing p53 expression in OCI-M1 cells treated with DNR (0.25 µM) with or without EPO (1 U/ml) for 6 h. p53 protein was detected with the p53 monoclonal antibody DO-1. **(c)** OCI-M1 cells were treated with DNR (0.25 µM) in the presence or absence of EPO (1 U/ml) for 24 h. The proportion of apoptotic cells (cells with < 2 N DNA content) was determined by flow cytometry after propidium iodide staining. Error bars represent SEM (N=3 biological replicates).
